# Vitamin D_3_ treatment differentially affects anxiety-like behavior in the old ovariectomized female rats and old ovariectomized female rats treated with low dose of 17β-estradiol

**DOI:** 10.1186/s12881-019-0774-2

**Published:** 2019-04-09

**Authors:** Julia O. Fedotova

**Affiliations:** ITMO University, Saint-Petersburg, Russia

**Keywords:** Vitamin D_3_, Anxiety, Menopause, Estradiol, Age, Old female rats

## Abstract

**Background:**

Estrogen deficiency effects on affective-related behavior are restricted to certain periods of age after ovary removal. Among other nutraceuticals, one of such «natural» substances for treatment of affective-related diseases could be vitamin D_3_. It is a great interest to evaluate the effects of repeated cholecalciferol administration on anxiety-related behavior in the old female rats with long-term estrogen deficiency. The present study was performed to determine the behavioral effects of cholecalciferol treatment at different doses as an adjunctive therapy alone or in a combination with low dose of 17β-estradiol on anxiety-like behavior of the old (16–18 months) female rats at 12 weeks after ovariectomy.

**Methods:**

Vitamin D_3_ supplementation individually (as cholecalciferol at doses of 1.0, 2.5 or 5.0 mg/kg/day, s.c.) or in co-administration with of 17β-estradiol (17β-E_2_, 0.5 μg/rat, s.c.) were given to the old ovariectomized (OVX) rats at 12 weeks after ovariectomy. Anxiety-related state was tested in the elevated plus maze (EPM) and light-dark test (LDT), as well behavioral reactivity was registered in the open field test (OFT). Moreover, 25-hydroxyvitamin D_3_ levels in the blood serum of these OVX rats treated with Vitamin D_3_ or Vitamin D_3_ plus 17β-E_2_ were measured.

**Results:**

The results of the present study indicated that Vitamin D_3_ supplementation at dose of 1.0 mg/kg/day decreased manifestations of anxiety-like profile in the old OVX rats. Treatment with Vitamin D_3_ (1.0 mg/kg/day) plus 17β-E_2_ in resulted in more profound anxiolytic-like effects the old OVX rats than effects of both drugs administered alone. Moreover, treatment with cholecalciferol (1.0 mg/kg/day, s.c.) in the old ovariectomized rats after ovariectomy at 12 weeks produced elevated estradiol and 25-OH-VD_3_ levels for these rats as compared to the old OVX females treated with oil solvent.

**Conclusions:**

Using the preclinical study, chronic cholecalciferol, 17β-E_2_ and their combination treatment were shown to be effective for anxiety-like treatment in the old subjects with long-term estrogen deficiency.

## Background

Anxiety disorders are twice common in women than in men and the risk is increased during the menopausal transition more [1, 2]. Menopause is a phase of a woman’s life which indicates termination of her reproductive function. It is induced by the loss of ovarian follicular activity, which leads to the permanent cessation of menstruation [2, 3]. Menopause is often associated with an onset of mood disturbances, especially anxiety and depression [3, 4]. For women, data suggest that estrogens are strongly implicated in the regulation of mood and behavior, as well as in the pathophysiology of mood disorders [5–7]. Nowadays, there is a strong tendency to rising of duration of living in the whole world, however, the median age of programmed termination of a woman’s reproductive life failed to altered [5–7]. Thus, majority of women in the ageing population stays for a significant period of their lifetime in a postmenopausal state which is clearly associated with very low estrogen levels that could one of the marked trigger factors for development of affective-related disorders [5, 7].

A strategy to alleviate the mood disorders associated with menopause is hormonal replacement therapy (HRT) [8]. However, controversial results related to the effectiveness of such treatment have been frequently reported [9]. These discrepancies could be associated to various factors, one of them being the time when estrogen restitution is initiated after the beginning of menopause [10, 11].

There is growing interest about the potential of diet and nutrients to improve the mental health of the women population and for the treatment of psychiatric disorders [10, 11]. In the case of mood disorders, the limitations of psychotropic drugs to achieve adequate rates of clinical remission and functional recovery have promoted the search for complementary approaches [12–15]. Menopausal women are now choosing to take alternative and complementary therapies marketed as «natural» treatments that offer the positive health effects of estrogens without the unwanted side effects [12, 13]. Effectiveness of α-tocopherol, ascorbic acid, β-carotene were previously shown for wide group of disorders and forms of administration [16, 17]. Among other nutraceuticals, one of such «natural» substances for treatment of affective-related diseases could be vitamin D (VD) [14, 15].

VD is a neuroactive secosteroid with well-known physiological role in skeleton formation and maintenance, as well as diverse «non-skeletal» functions [18, 19]. «Non-skeletal» functions of VD are connected with its different range of outcomes in the central nervous system, such as neuroplasticity, apoptosis, cell proliferation and differentiation [20, 21]. All functions of VD in the body (classical functions, i.e., effect upon calcium-phosphate management and the non-classical ones) are imposed by the VD nuclear receptor (VDR), regulating directly the gene expression [22, 23]. Nuclear VDR are member of receptors family for transcription factors which are activated by numerous ligands [22, 23]. VDR are present in most tissues and cells in the body, and within the brain show some specificity to the prefrontal cortex, hippocampus, cingulate gyrus, thalamus, hypothalamus and substantia nigra [24]. This is of relevance as many of those brain regions have been implicated in the physiology of affective-related disorders.

Estrogen deficiency effects on affective-related behavior are restricted to certain periods of age after ovary removal [25–27]. Preclinical data suggest that onset age of menopause can be important to obtain behavioral positive or negative results. Thus, it is a great interest to evaluate the effects of repeated cholecalciferol administration on anxiety-related behavior in the middle-aged and old female rats with long-term estrogen deficiency.

The aim of the present study was to determine if repeated systemic treatment with cholecalciferol affected on anxiety-like behavior in the old female rats after long-term ovariectomy.

## Methods

### Animals

Female albino Wistar rats (16–18 months, old rats, weighing 260–270 g, respectively) from the special biocollection of Koltushi vivarium (St. Petersburg, Russia) were used in the present study. All rats were allocated in groups and were allowed to accommodate for 1 week in the animal house at I.P. Pavlov Institute of Physiology, of the Russian Academy of Sciences, before subjecting them to behavioral testing and pharmacological treatments. They were provided with a standard pellet diet and were given water ad libitum. The animals were kept at a temperature of 23 °C ± 2 °C and a 12 h light/dark cycle as well as a constant relative humidity (50% ± 10%) during all experimental sessions. Female rats of different age rats were randomly separated into experimental groups 4, including the control groups.

Vitamin D_3_ and 17β-E_2_ treatments, as well as anxiety-related tests were carried out a double-blind method by using rules of the Health guide for the care and use of Laboratory animals (1978) formulated by the National Institute of Health. Females of different age were placed to the special room for behavioral trials at least 1 h prior to the beginning of the experimental sessions which were performed from 09:00 am to 12:00 am. The experimental protocols of this study were approved by the Institutional Animal Ethics Committee of I.P. Pavlov Institute of Physiology, Russia (protocol 1095/1 from June 25, 2012).

### Surgery

Long-term ovariectomy surgery was performed as previously described [28]. Briefly, middle-aged and old female rats were anesthetized with ketamine (70 mg/kg b.w.) mixed with xylazine (10 mg/kg b.w.). To avoid inflammation, the rats were administered with meloxicam (1 mg/kg b.w.). The fallopian tube was crushed and the ovary was removed by cutting. The effectiveness of long-term ovariectomy or 17β-estradiol (17β-E_2_) application was assessed by vaginal smears. The old ovariectomized (OVX) females were housed in groups of five in cages separated by groups. To assure the long-term absence of estrogens, all rats after surgery were remained to the housing facilities for 12 weeks.

### Drug treatments

17β-Estradiol, 17β-E_2_ (Sigma, USA) at low dose of 5.0 μg/rat [27, 28] and Vitamin D_3_ as cholecalcirefol (Sigma, USA) at several doses (1.0, 2.5 or 5.0 mg/kg) [29] were subcutaneously (s.c.) administered once daily starting 14 days prior to the cognitive experiments. 17β-E_2_ was dissolved in sterile sesame oil, VD_3_ – in 95% ethanol solvent, aliquoted and stored at -80 °C. The stock of VD_3_ was dissolved in a sterile water, resulting in a solution of cholecalciferol with 2% ethanol. All drug solutions were freshly prepared before each behavioral testing. 17β-E_2_ and cholecalcirefol were injected in a volume of 0.1 ml. The estrogen, 17β-E_2_ (E-8875, Sigma Chemical Co., St. Louis, MO, USA) was dissolved in sterile sesame oil. Cholecalcirefol (C-9756, Sigma Chemical Co., St. Louis, MO, USA) was dissolved in 95% ethanol, aliquoted and stored at -80 °C. The stock of cholecalciferol was diluted in a sterile water, resulting in a solution of cholecalciferol with 2% ethanol. 17β-E_2_ was injected subcutaneously (s.c. at a dose of 0.5 μg/rat). The low dose of 17β-E_2_ (5.0 μg/rat subcutaneously, s.c.) was chosen from the studies performed by Estrada-Camarena and co-workers [29, 30]. Three doses of cholecalciferol (1.0, 2.5 or 5.0 mg/kg, s.c.) were chosen from the behavioral study performed by Idrus and co-workers [31]. All solutions were freshly prepared before each experimental series. All preparations were administered in a volume of 0.1 ml. Following 12 weeks after ovariectomy, cholecalciferol, 17β-E_2_ and oil solvent were injected once daily for 14 days.

### Animal groups

Female rats (old intact and OVX) were randomly divided into 24 groups, accordingly to their age, with 8 rats in each group.

The following experimental groups for the old female rats were created in the present study:

1. old intact female rats + solvent,
2. old intact female rats + cholecalciferol 1.0 mg/kg (old intact-Vit D_3_ 1.0),
3. old intact female rats + cholecalciferol 2.5 mg/kg (old intact-Vit D_3_2.5),
4. old intact female rats + cholecalciferol 5.0 mg/kg (old intact-Vit D_3_ 5.0),
5. old OVX + solvent (old OVX-Sol),
6. old OVX rats +17β-E_2_ (old OVX-17β-E_2_),
7. old OVX rats + cholecalciferol 1.0 mg/kg (old OVX-Vit D_3_ 1.0),
8. old OVX rats + cholecalciferol 2.5 mg/kg (old OVX-Vit D_3_ 2.5),
9. old OVX rats + Vit D_3_ 5.0 mg/kg (old OVX-Vit D_3_ 5.0),
10. old OVX rats + cholecalciferol 1.0 mg/kg + 17β-E_2_ (old OVX-Vit D_3_ 1.0-17β-E_2_),
11. old OVX rats + cholecalciferol 2.5 mg/kg + 17β-E_2_ (old OVX-Vit D_3_ 2.5-17β-E_2_),
12. old OVX rats + cholecalciferol 5.0 mg/kg + 17β-E_2_ (old OVX-Vit D_3_ 5.0-17β-E_2_).

The treatment period for animals was 14 days, and at the end of the treatment period (1 h after the last dose of solvent, Vitamin D_3_ or 17β-E_2_), all animals were subjected to the EPM, LDT and the open field test. During testing sessions in all behavioral tests the control and experimental groups of rats were also given with solvent, Vitamin D_3_ or 17β-E_2._

### Behavioral tests

#### Elevated plus maze test

EPM is a commonly accepted as standard test of anxiety-like behavior and was used to assess an anxiety-like behavioral responses [32, 33]. This test is sensitive to putative anxiogenic-like and anxiolytic-like drugs [34]. The EPM is consisted of two open arms (50 × 10 cm2) and two closed arms (40 × 10 cm2) with a central platform (10 × 10 cm2), and elevated 50 cm above the floor level. All female rats from control and experimental groups were randomly placed at the center of the EPM and allowed them to freely move in the apparatus for 5 min. The number of entries and total time spent in open arms were accepted as parameters of anxiolytic-like effects of treatments. The apparatus was cleaned with damp cloth after each trial to avoid place preference and the influence of olfactory stimuli.

#### Light/dark test

The apparatus was consisted of two identical boxes (30 × 40 × 40 cm), one of which with white walls and floor and illuminated by a 60 W light from above, while the other of the box was painted black and had a lid so it was not illuminated [35, 36]. The number of entrances and the total time in the light box were registered for 5 min [7]. The increase of the number of entrances and the total time in the light box were postulated as manifestation of anxiolytic-like effects of treatments. The apparatus was cleaned with damp cloth after each trial to avoid place preference and the influence of olfactory stimuli.

#### Open field test

The effect of cholecalciferol on locomotor, rearing and grooming activities was evaluated automatically using an open-field computer-aided controlling system as described previously [37]. The apparatus consists a square platform (80.0 cm × 80.0 cm; wall height 36.0 cm). The floor of the platform was divided into 16 equal squares of 19.5 cm × 19.5 cm. A video camera fixed at the top, and the apparatus was illuminated by a light source of 120 Lux on the ceiling. Each rat was placed at the center of the apparatus and allowed to explore freely for 5 min. Total number of central and peripheral square crossings were recorded for each animal. The apparatus was cleaned with damp cloth after each trial to avoid place preference and the influence of olfactory stimuli.

### Determination of estradiol, 25-OH-VD_3_ and calcium levels in the blood serum

Blood samples were collected in tubes and centrifuged. After centrifugation, serum was separated, frozen and stored at − 20 °C until biochemical assessment. Estradiol levels was assessed using commercial available ELISA kit (DRG Diagnostics, Marburg, Germany). The sensitivity for estradiol detection using ELISA kit was 3.0 pg/ml. Measurement of 25-hydroxyvitamin D_3_ (25-OH-VD_3_) levels was performed by ELISA kit (CSB-E08098r, Cusabio Biotech Co., Ltd., Wuhan, P.R. China). Technical variability for 25-OH-VD_3_ ELISA kit was low with coefficients of variation of < 10% intra-assay and < 15% inter-assay. Detection range of 25-OH-VD_3_ levels was 20 μg/L-100 μg/L. The sensitivity of the 25-OH-VD_3_ ELISA kit was 5.0 μg/L. Calcium concentrations were detected by spectrophotometric method using Calcium assay colorimetric kit (ab102505, Abcam, France). The sensitivity of the calcium kit was 0.1 m/M. All the procedures of estradiol, 25-OH-VD_3_ and calcium kits were conducted following the manufacturer’s instruction manual.

### Statistical analysis

Data were expressed as means ± standard error (S.E.M). Differences among means were postulated as significant at р ≤ 0.05. Behavioral and biochemical data were analyzed using a two-way ANOVA and subsequent post hoc analysis with Dunnett’s multiple comparison test. Statistical calculation was carried out using SPSS software 19 version (SPSS Inc., Chicago, IL., USA).

## Results

### Vitamin D_3_ in different doses decreases anxiety-like profile of the old OVX and OVX rats given with 17β-estradiol after long-term absence of estrogen as measured in the EPM test

Two-way ANOVA test demonstrated a significant hormone condition × treatment interaction ([F(5,44) = 11.44, *P* < 0.05] and [F(5,44) = 11.12, *p* < 0.01], respectively), significant effect of hormone conditions ([F(5,44) = 9.22, *P* < 0.01] and [F(5,44) = 16.88, *p* < 0.01], respectively) and significant effect for treatment ([F(5,44) = 11.56, *P* < 0.05], and [F(5,44) = 12.56, *p* < 0.05], respectively) for the time spent into the open arms or the number of entries into the open arms of the old OVX rats. Post-hoc analyses revealed differences among the groups for the anxiety-like state during experimental sessions (*p* < 0.05).

**Fig. 1:**
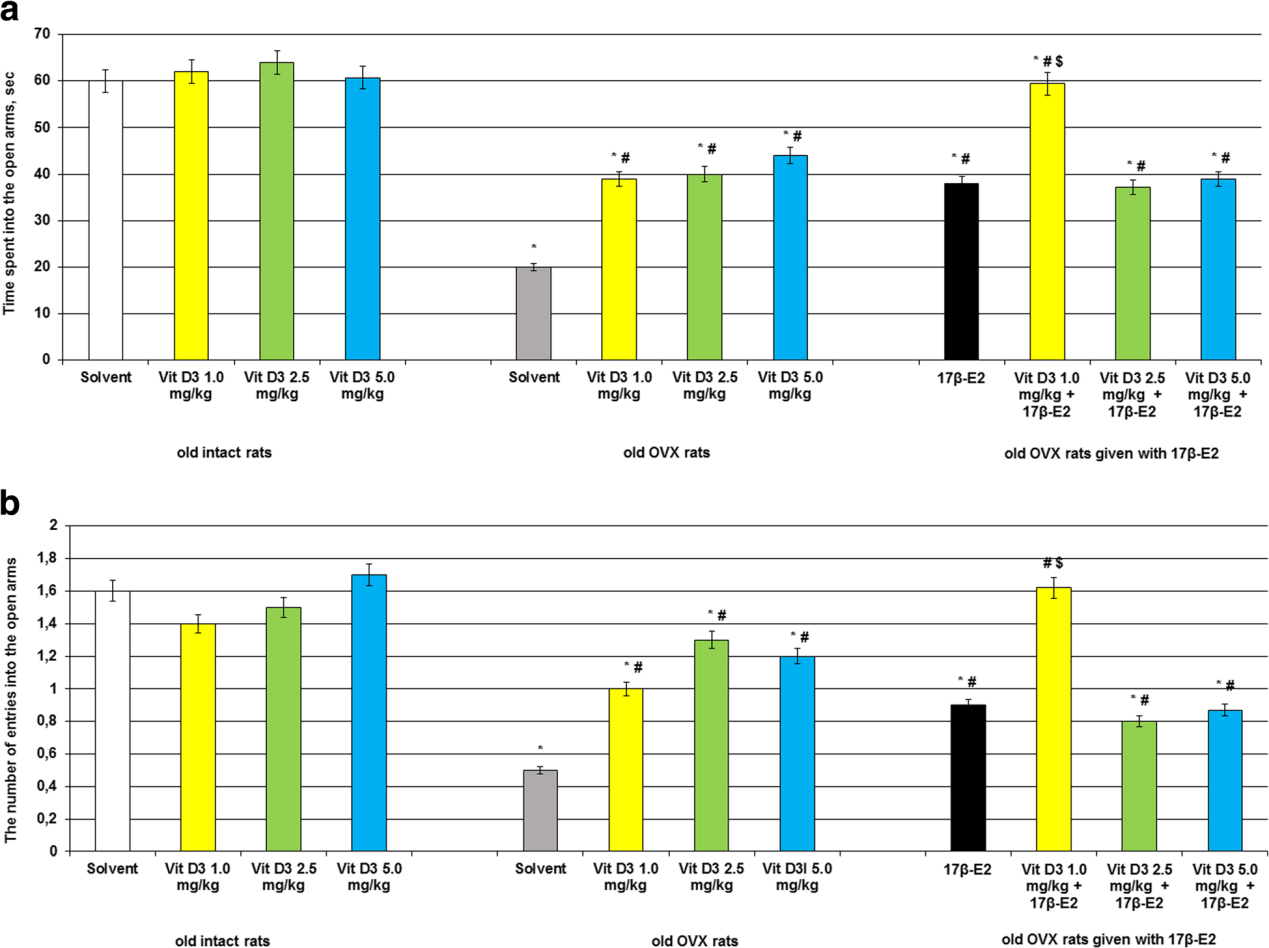
Effects of Vitamin D_3_ administration on anxiety-like behavior of the old ovariectomized (OVX) rats following long-term estrogen deficiency in the elevated plus maze. **a** – time spent into the open arms, sec; **b** – the number of entries into the open arms. The obtained results show the mean ± standard error of the mean (SEM). *– *p* < 0.05 as compared to the control group of the old sham-operated rats; # – *p* < 0.05 as compared to the old OVX rats treated with solvent; $ – *p* < 0.05 as compared to the old OVX rats treated with 17β-estradiol (17β-E_2_). Each group comprised a minimum of eight rats. Cholecalciferol was given at 1.0, 2.5 or 5.0 mg/kg/day subcutaneously (s.c.), once daily, for 14 days. The administered dose of 17β-estradiol was 0.5 μg/rat s.c., once daily, for 14 days

The old intact rats treated with Vitamin D_3_ at doses of 1.0 mg/kg, 2.5 mg/kg or 5.0 mg/kg failed to alter the time spent into the open arms and the number of entries into the open arms as compared to the old control rats (Fig. 1ab, *p* > 0.05). The old OVX rats given with solvent displayed a significant decrease of the time spent into the open arms and the number of entries into the open arms as compared to the old control rats (Fig. 1ab, *p* > 0.05).

Vitamin D_3_ supplementation administered in all doses to the old OVX rats resulted in increase of the time spent into the open arms and the number of entries into the open arms as compared to the old OVX rats treated with solvent (Fig. 1ab, *p* > 0.05). Combined administration of cholecalciferol at dose of 1.0 mg/kg with 17β-E_2_ to the old OVX rats more significantly increased the time spent into the open arms and the number of entries into the open arms as compared to the old OVX rats treated with solvent or 17β-E_2_ (Fig. 1ab, *p* > 0.05). The old OVX rats administered with cholecalciferol at doses of 2.5 mg/kg and 5.0 mg/kg plus 17β-E_2_ showed similar values of the time spent into the open arms and the number of entries into the open arms like as old OVX rats treated with 17β-E_2_ (Fig. 1ab, *p* > 0.05).

### Vitamin D_3_ at different doses reverses anxiety-like profile of the old OVX and OVX rats given with 17β-estradiol after long-term absence of estrogen as measured in light-dark box

Two-way analysis of variance showed a significant hormone condition × treatment interaction ([F(5,44) = 11.52, *p* < 0.01]), significant effect of hormone conditions ([F(5,44) = 11.22, *p* < 0.05]) and significant effect for treatment ([F(5,44) = 10.08, p < 0.01]) for time spent and number of entries in the light compartment of the old OVX rats. Post-hoc analyses revealed differences among the groups for the anxiety-like state during experimental sessions (*p* < 0.05).

**Fig. 2:**
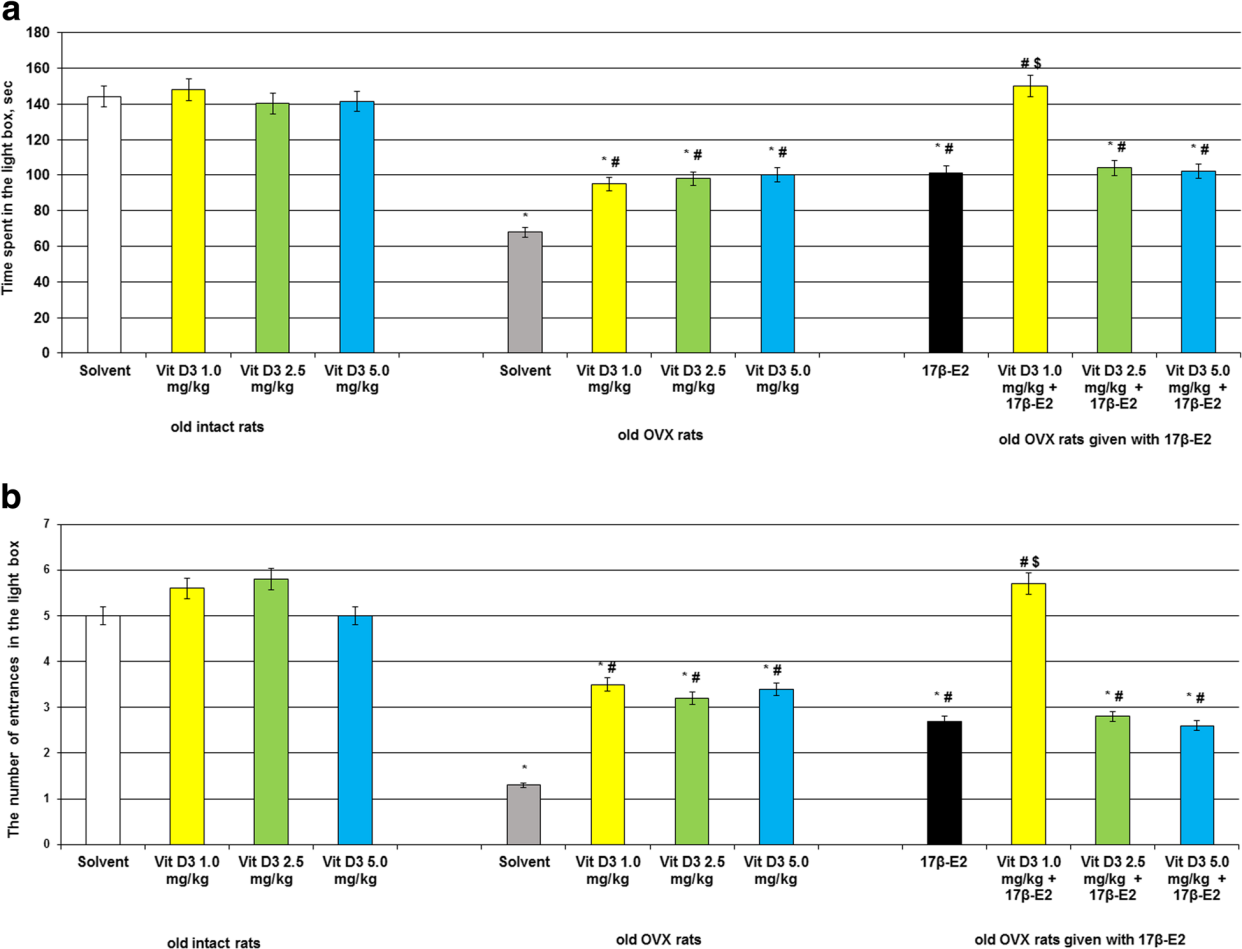
Effects of Vitamin D_3_ administration on anxiety-like behavior of the old OVX rats following long-term estrogen deficiency in the light/dark test. **a** – time spent in the light box, sec; **b** – the number of entrances in the light box. The obtained results show the mean ± standard error of the mean (SEM). * – *p* < 0.05 as compared to the control group of sham-operated rats, # – *p* < 0.05 as compared to the old OVX rats treated with solvent, $ – *p* < 0.05 as compared to the old OVX rats treated with 17β-estradiol. Each group comprised a minimum of eight rats. Cholecalciferol was given at 1.0, 2.5 or 5.0 mg/kg/day s.c., once daily, for 14 days. The administered dose of 17β-estradiol (17β-E_2_) was 0.5 μg/rat s.c., once daily, for 14 days

The old intact rats treated with Vitamin D_3_ at doses of 1.0 mg/kg, 2.5 mg/kg or 5.0 mg/kg failed to modify time spent and number of entries in the light compartment as compared to the old control rats (Fig. 2ab, *p* > 0.05). The old OVX rats given with solvent displayed a significant decrease of time spent and number of entries in the light compartment as compared to the old control rats (Fig. 2ab, *p* > 0.05).

Vitamin D_3_ supplementation administered in all doses to the old OVX rats induced increase of the time spent and number of entries in the light compartment as compared to the old OVX rats treated with solvent (Fig. 2ab, *p* > 0.05). Combined administration of cholecalciferol at dose of 1.0 mg/kg with 17β-E_2_ to the old OVX rats more significantly increased time spent and number of entries in the light compartment as compared to the old OVX rats treated with solvent or 17β-E_2_ (Fig. 2ab, *p* > 0.05). The old OVX rats administered with Vitamin D_3_ at doses of 2.5 mg/kg and 5.0 mg/kg plus 17β-E_2_ showed identical parameters of time spent and number of entries in the light compartment like as old OVX rats treated with 17β-E_2_ (Fig. 2ab, *p* > 0.05).

### Vitamin D_3_ administration changes behavioral reactivity of the old OVX and OVX rats treated with 17β-estradiol

Accordingly to the two-way ANOVA test, there were significant differences for the grooming behavior between hormone conditions ([F(5,44) = 8.12, *p* < 0.05]) between drug treatment ([F(5,44) = 12.51, *p* < 0.01]) and an interaction between hormone condition and treatments ([F(5,44) = 7.16, *p* < 0.01]) in the old OVX rats. Further *post-hoc* test revealed differences for grooming between experimental groups of the old OVX rats (*p* < 0.05).

Vitamin D_3_ injected at several doses failed to demonstrate any changes of behavioral reactivity of the old intact females in the OFT as compared to the control rats (Table 1, *p* > 0.05).

**Table 1:** Effects of Vitamin D_3_ administration on behavior of the old ovariectomized (OVX) rats following long-term estrogen deficiency in the open field test for 5 min

Groups	Crossing	Rearing	Grooming
Old control rats + solvent	69.7 ± 5.2	11.5 ± 0.3	2.9 ± 0.2
Old intact rats + cholecalciferol 1.0 mg/kg	71.8 ± 2.9	10.7 ± 0.3	3.2 ± 0.2
Old intact rats + cholecalciferol 2.5 mg/kg	66.9 ± 3.6	10.4 ± 0.2	3.1 ± 0.2
Old intact rats + cholecalciferol 5.0 mg/kg	69.0 ± 4.2	12.2 ± 0.8	3.0 ± 0.2
Old OVX rats + solvent (OVX/solvent rats)	72.1 ± 2.3	12.1 ± 0.6	1.0 ± 0.2*
Old OVX rats +17β-E_2_ (OVX/17β-E_2_ rats)	64.3 ± 4.6	11.7 ± 0.8	3.1 ± 0.3^#^
Old OVX rats + cholecalciferol 1.0 mg/kg	63.2 ± 3.5	12.6 ± 0.9	4.2 ± 0.2^#^
Old OVX rats + cholecalciferol 2.5 mg/kg	67.2 ± 5.2	10.2 ± 0.8	3.9 ± 0.2^#^
Old OVX rats + cholecalciferol 5.0 mg/kg	70.3 ± 4.4	11.5 ± 0.5	4.3 ± 0.2^#^
Old OVX rats + cholecalciferol 1.0 mg/kg + 17β-E_2_	72.1 ± 6.8	12.2 ± 0.6	0.7 ± 0.2* ^# $^
Old OVX rats + cholecalciferol 2.5 mg/kg + 17β-E_2_	78.5 ± 8.4	11.8 ± 0.4	0.6 ± 0.2* ^# $^
Old OVX rats + cholecalciferol 5.0 mg/kg + 17β-E_2_	69.4 ± 6.6	10.9 ± 0.8	0.9 ± 0.2* ^# $^

The obtained results show the mean ± standard error of the mean (SEM). *p < 0.05 as compared to the control group of the old sham-operated rats; ^#^*p* < 0.05 as compared to the old OVX rats treated with solvent; ^$^*p* < 0.05 as compared to the old OVX rats treated with 17β-estradiol (17β-E_2_). Each group comprised a minimum of eight rats. Cholecalciferol was given at 1.0, 2.5 or 5.0 mg/kg/day subcutaneously (s.c.), once daily, for 14 days. The administered dose of 17β-estradiol was 0.5 μg/rat s.c., once daily, for 14 days

A significant decrease of grooming behavior was registered in the old OVX rats given with solvent as compared to the control (Table 1, *p* < 0.05). 17β-E_2_ significantly reduced grooming reactions in the old OVX rats as compared to the middle-aged OVX rats (Table 1, *p* < 0.05). The old OVX rats treated with Vitamin D_3_ in all tested doses alone or in a combination with 17β-E_2_ did not demonstrate any modifications of motor and rearing activities as compared to the OVX rats given with solvent (Table 1, *p* < 0.05).

However, the old OVX rats treated with Vitamin D_3_ at doses of 1.0, 2.5 and 5.0 mg/kg demonstrated an increase of grooming behavior as compared to the OVX rats. A co-administration of Vitamin D_3_ at these doses with 17β-E_2_ decreased grooming behavior as compared to old intact and OVX rats received with solvent or 17β-E_2_ (Table 1, *p* < 0.05).

### Modifications of 25-hydroxyvitamin D_3_, estradiol and calcium levels in the blood serum following Vitamin D_3_ administration in the old OVX and OVX females treated with 17β-estradiol

**Fig. 3:**
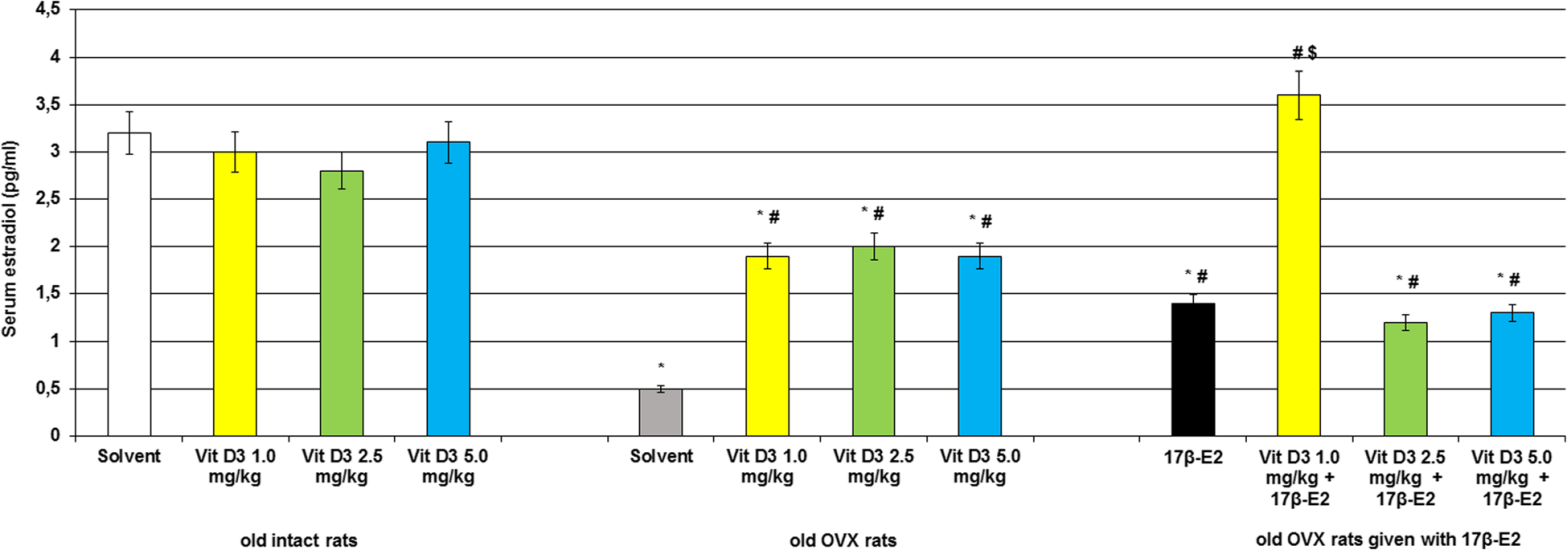
Effects of Vitamin D_3_ administration on estradiol level of the old ovariectomized (OVX) rats following long-term estrogen deficiency in the serum blood. The obtained results show the mean ± standard error of the mean (SEM). *– *p* < 0.05 as compared to the control group of the old sham-operated rats; # – *p* < 0.05 as compared to the old OVX rats treated with solvent; $ – *p* < 0.05 as compared to the old OVX rats treated with 17β-estradiol (17β-E_2_). Each group comprised a minimum of eight rats. Cholecalciferol was given at 1.0, 2.5 or 5.0 mg/kg/day subcutaneously (s.c.), once daily, for 14 days. The administered dose of 17β-estradiol was 0.5 μg/rat s.c., once daily, for 14 days

**Fig. 4:**
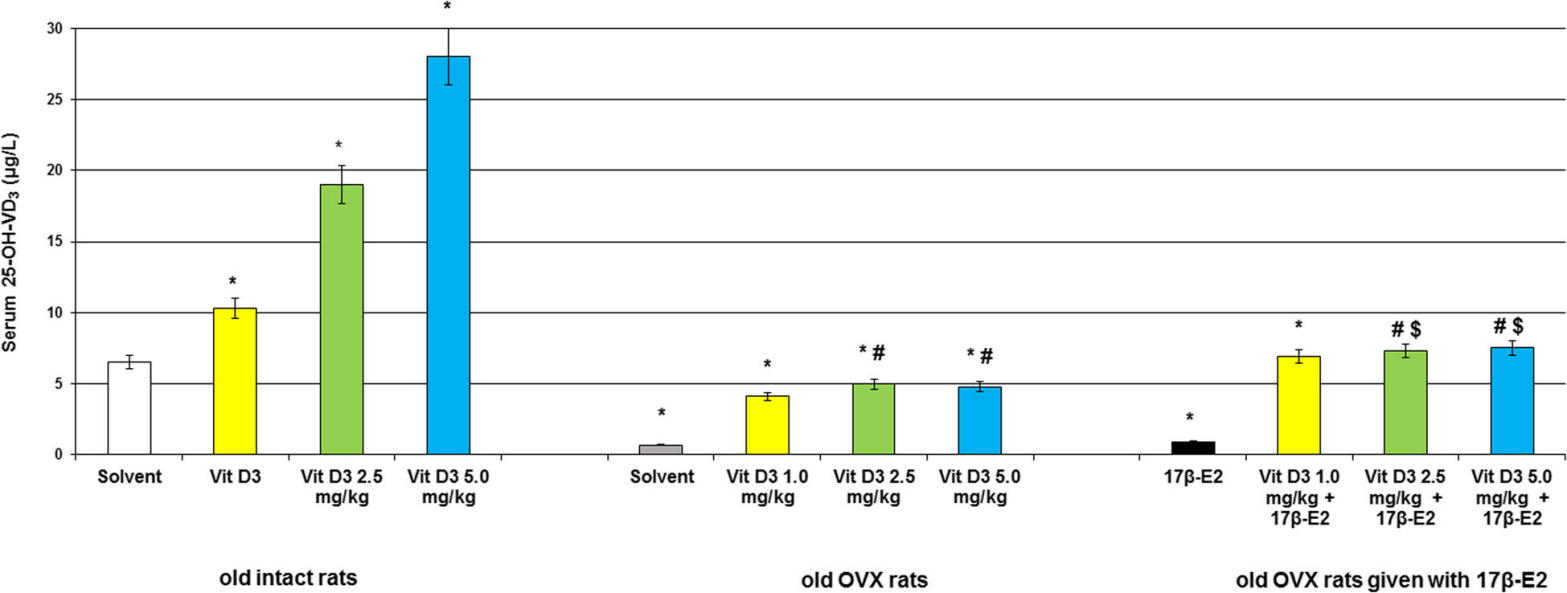
Effects of Vitamin D_3_ administration on 25-OH-VD_3_ level of the old ovariectomized (OVX) rats following long-term estrogen deficiency in the serum blood. The obtained results show the mean ± standard error of the mean (SEM). *– *p* < 0.05 as compared to the control group of the old sham-operated rats; # – *p* < 0.05 as compared to the old OVX rats treated with solvent; $ – *p* < 0.05 as compared to the old OVX rats treated with 17β-estradiol (17β-E_2_). Each group comprised a minimum of eight rats. Cholecalciferol was given at 1.0, 2.5 or 5.0 mg/kg/day subcutaneously (s.c.), once daily, for 14 days. The administered dose of 17β-estradiol was 0.5 μg/rat s.c., once daily, for 14 days

The old intact rats treated with cholecalciferol at doses of 1.0, 2.5 and 5.0 mg/kg failed to alter estradiol levels in the serum blood as compared to the control rats (Fig. 3, *p* > 0.05) and increased 25-OH-VD_3_ levels (Fig. 4, *p* < 0.05). Long-term ovariectomy in the old female rats resulted in a significant decrease of estradiol and 25-OH-VD_3_ levels in the blood as compared to the old control females (Figs. 3 and 4, *p* < 0.05). The 17β-E_2_ supplementation (0.5 μg/kg, s.c.) failed to modify 25-OH-VD_3_ levels in the blood of the old OVX rats as compared to the old OVX rats administered with solvent (Fig. 4, *p* > 0.05), and the value of this parameter in the old OVX/17β-E_2_ females were lower than that of the value of old control rats. However, 17β-E_2_ supplementation significantly increased estradiol levels in the blood of the old OVX rats as compared to the old OVX rats given with solvent (Fig. 3, *p* < 0.05).

The old OVX rats treated with cholecalciferol at all doses significantly increased 25-OH-VD_3_ and estradiol levels in the serum blood as compared to the old OVX rats treated with solvent (Fig. 4, *p* < 0.05). However, the values of 25-OH-VD_3_ and estradiol levels in the old OVX rats treated with cholecalciferol at all doses were lower than that of the values of old control rats.

Co-administration of Vitamin D_3_ (1.0 mg/kg) and 17β-E_2_ markedly enhanced estradiol levels in the old OVX rats as compared to the groups of old OVX rats received with solvent or 17β-E_2_ (Fig. 3, p < 0.05). Vitamin D_3_ supplementation (2.5 mg/kg and 5.0 mg/kg) plus 17β-E_2_ did not modify estradiol concentrations in the serum blood of the old OVX rats as compared to the OVX rats given with 17β-E_2_ (Fig. 3, *p* > 0.05). Cholecalciferol at all doses in combination with 17β-E_2_ significantly increased 25-OH-VD_3_ levels when these old OVX rats were compared with the old OVX/solvent and OVX/17β-E_2_ groups (Fig. 4, *p* > 0.05).

**Fig. 5:**
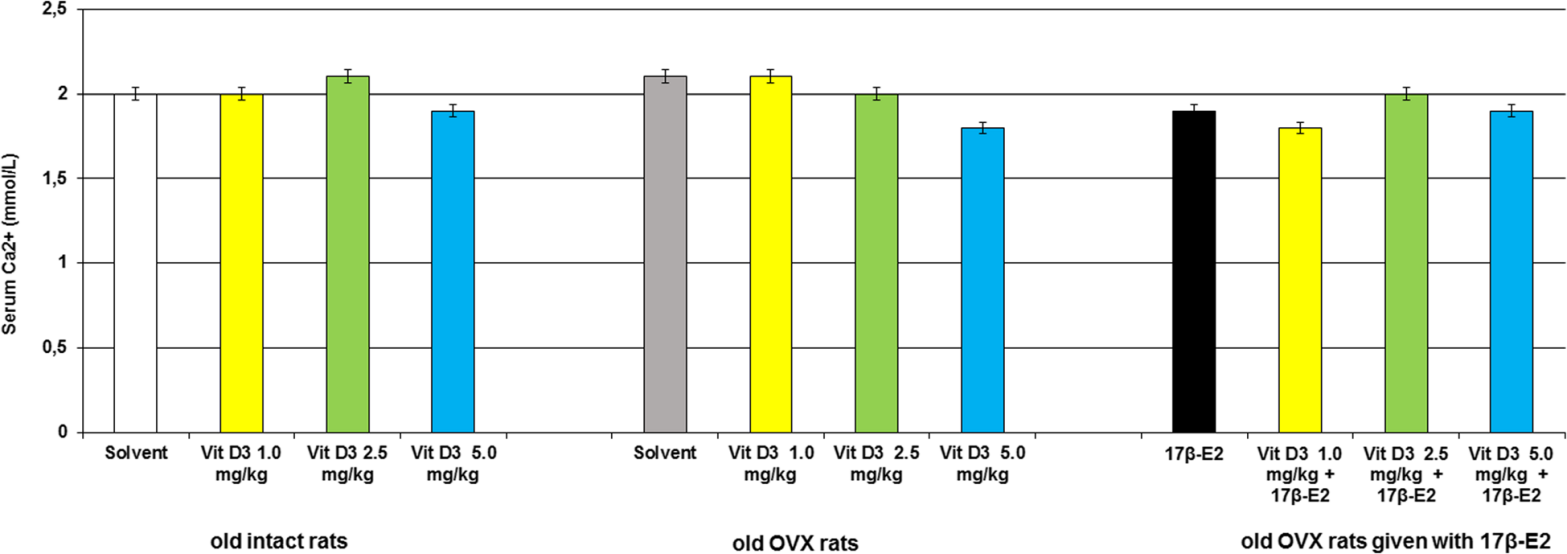
Effects of Vitamin D_3_ administration on calcium level of the old ovariectomized (OVX) rats following long-term estrogen deficiency in the serum blood. The obtained results show the mean ± standard error of the mean (SEM). *– *p* < 0.05 as compared to the control group of the old sham-operated rats; # – *p* < 0.05 as compared to the old OVX rats treated with solvent; $ – *p* < 0.05 as compared to the old OVX rats treated with 17β-estradiol (17β-E_2_). Each group comprised a minimum of eight rats. Cholecalciferol was given at 1.0, 2.5 or 5.0 mg/kg/day subcutaneously (s.c.), once daily, for 14 days. The administered dose of 17β-estradiol was 0.5 μg/rat s.c., once daily, for 14 days

The two-way ANOVA failed to show any significant differences in the calcium levels in the blood serum between hormone conditions, drug treatments, and an interaction between hormone condition and treatments in the old OVX rats with long-term estrogen deficiency (Figs. 5, *p* > 0.05). The post-hoc test did not find any differences among the experimental groups for the calcium levels (*p* > 0.05).

## Discussion

In the present study, the effects of chronic cholecalciferol treatment at different doses (1.0, 2.5 and 5.0 mg/kg, s.c.) for 14 days on anxiety-like behavior in the old female rats with long-term estrogen deficiency and 17β-E_2_ supplementation in a low dose were examined. Cholecalciferol at all investigated doses did not produce any changes of anxiety-like behavior of the old intact female rats in the EPM and LDT. Analyzing the results from biochemical assay, an increase of 25-OH-VD_3_ concentrations and absence of any modifications of estradiol levels in the serum blood of the middle-aged and old intact rats given with different doses of cholecalciferol were found.

These results suggest that cholecalciferol-induced the increasing of 25-OH-VD_3_ levels in the blood serum of the old intact-ovary rats are not associated with absence of anxiety-like profile alterations in the behavioral tests. Furthermore, the old ovary-intact female rats are also needed to evaluate the behavioral effects of cholecalciferol administered at several doses in the EPM and LDT paradigms.

The results showed that in the old OVX rats following 12 weeks of postovariectomy period, there were marked anxiety-like behavior as assessed by EPM and LDT. Although 17β-E_2_ supplementation resulted in significant anxiolytic-like effect of the old OVX rats with long-term absence of estrogen, the 17β-E_2_ administration was not able to completely diminish anxiety-like behavior to the level of the old control intact animals. According to these results, we conclude that old OVX rats following 12 weeks of postovariectomy period display significant anxiety-related behavior, while 17β-E_2_ administration to the old OVX rats attenuates the estrogen deficiency-induced anxiety-like behavior to some extent. In fact, these experiments showed that the effects of 17β-E_2_ supplementation on anxiety-like behavior did not associated with absence of its effects on 25-OH-VD_3_ levels in the old OVX rats. The long-term effect of ovariectomy on anxiety-like behavior in old female rats were submitted in a standard behavioral tests [26, 27, 38].

Cholecalciferol at all doses per se had a significant anxiolytic-like effect in the old OVX rats following long-term ovariectomy. Unexpectedly, that the old OVX rats with 12 weeks postovariectomy period administered with cholecalciferol at doses 2.5 and 5.0 mg/kg/day in combination with 17β-E_2_ showed similar anxiety-like profile like the OVX rats given with 17β-E_2_. Thus, we did not observe any synergic anxiolytic-like effects of cholecalciferol at doses of 2.5 mg/kg or 5.0 mg/kg in the old OVX rats given with 17β-E_2._ It might supposed that there are some concurrent relation between 17β-E_2_ and cholecalciferol at doses of 2.5 and 5.0 mg/kg/day. In fact, application of 17β-E_2_ interfere with anxiolytic-like action of cholecalciferol at doses of 2.5 mg/kg or 5.0 mg/kg in the old OVX rats after long-term ovariectomy. Simultaneously, cholecalciferol treatment in all tested doses similarly increased grooming, did not change locomotor activity and rearing of the old OVX rats after long-term ovariectomy. Thus, the present results suggest that anxiolytic-like effects of Vitamin D_3_ are specific in the old OVX rats given with solvent or 17β-E_2_, since data of the OFT were not able to demonstrate any of alterations in motor or rearing activities in these rats.

Biochemical analysis showed that administration of cholecalciferol at all doses alone or in a combination with 17β-E_2_ resulted in elevated 25-OH-VD_3_ levels in the blood serum of the old OVX rats with long-term absence of estrogen. Cholecalciferol administered alone at all doses similarly increased estradiol levels in the blood serum of the old OVX rats after long-term ovariectomy. On the other hand, only application of cholecalciferol at a dose of 1.0 mg/kg with low dose of 17β-E_2_ induced more profound increase of estradiol levels in the blood serum of the old OVX rats. Moreover, cholecalciferol in several doses failed to induce any changes in calcium concentrations in the blood serum of the old OVX rats given with solvent or 17β-E_2_.

These data suggested that the different effects of cholecalciferol application per se in the old OVX rats with long-term absence of estrogen on anxiety-like behavior in the EPM and LDT did not associated with its effects on estradiol, 25-OH-VD_3_ and calcium levels.

However, we can speculate that behavioral effects of cholecalciferol treatment with low dose of 17β-E_2_ in the EPM and LDT tests might connected with fluctuations of estradiol levels in the blood serum of the old OVX rats. It is possible that specific sites of action involved in the anxiolytic-like effects of cholecalciferol that also modulated by estrogens are affected by the endocrine milieu that prevails at different period for the old female rats. Moreover, after a long-time absence of ovarian fluctuations an adaptive process may contribute to a better response for cholecalciferol administration at a dose of 1.0 mg/kg in the old female rats.

The role of ovarian hormones in anxiety and stress sensitivity is of great interest for women transitioning through menopause [39, 40]. Mood disorders during menopause could be partly due to loss of estrogen with menopause because estrogen is known to have neuroprotective effects on brain [41, 42]. Menopausal hormonal therapy (MHT) may improve the symptoms of affective-related state in people or decrease the risk of developing mood disturbances in older women, but this is unclear because in some studies MHT does not stop the development of anxiety-like symptoms in elderly postmenopausal women [43]. The exact role of estrogen still needs to be defined.

Menopause are also at higher risk of developing VD deficiency due to decreased dietary intake, less sun exposure, restricted outdoor activity and a decreased capacity to produce enough calcitriol as a result of an age related decline in hydroxylation by kidneys [44–46].

Vitamin D_3_ is a hormone precursor which is transformed into 1,25-dihydroxyvitamin D (1,25-(OH)_2_D_3_) in the liver and kidney [47]. Through decades This active form of VD has been involved in the brain development and functions of the central nervous system (CNS) [48, 49]. Hormonal form 1,25(OH)2D_3_ enters the brain via the blood brain barrier to act directly on cells containing its nuclear receptor, the VDR [50, 51].

The presence of VD receptors (VDR) outside the skeletal system, enterocytes and renal tubular cells was confirmed in many cell types including immune cells, neurons, pancreatic cells, myocytes, cardiomyocytes, endothelium cells, which stress pleiotropic activity of VD [52, 53]. The active form of vitamin D is transferred to astrocytes where it can bind to VDR and initiate gene transcription or be inactivated when in excess [50, 51]. Alternatively, 1,25(OH)_2_D_3_ can induce autocrine or paracrine rapid non-genomic actions since all brain cell types express the other membrane receptor of VD [50]. It is possible that the behavioral effects of cholecalciferol are mediated by multiple target regions, including brain centers that are involved in the mechanisms of anxiety-like behavior. Regardless, it cannot presently exclude possible indirect effects of vitamin D_3_ on different neurotransmitter circuits. Further research is required to investigate the underlying mechanisms in its anxiolytic-like effects.

It is well-established both systemic effects of VD on calcium metabolism and neuroprotective effects of VD on the brain [54]. Low VD levels has been implicated in the pathophysiological mechanisms of cardiovascular diseases, depression, anxiety, cognitive disorders, obesity, metabolic syndrome, type 2 diabetes, various types of cancer, immune disorders [10, 54]. According to Gaugris and co-workers (2005) [55], the prevalence of low VD levels appears to be high in postmenopausal women. Additionally, the decline of estrogens after menopause decreases the activity of 1α-OHase, what results in lower synthesis of the active VD form [55, 56]. Application of Vitamin D_3_ in specific periods of women’s life, seems to be of great importance, because it may reduce the risk of affective-related diseases during menopausal period [57–59]. However, the current data for Vitamin D_3_ application studies are very incomplete and need of more intensive investigations. The main point of question is to examine how the interaction between Vitamin D_3_ and estradiol might alter at specific periods of women’s life, and the impacts of such alterations elsewhere in the postmenopausal woman. VDR have been identified throughout the female reproductive tract [60, 61]. Some studies have demonstrated a direct modulation by VD of estradiol, estrone, and progesterone production in human ovarian cells [62–64]. It could be supposed that estrogens and VD share similar targets of the brain to induce their anxiolytic-like effects. However, the behavioral manifestations of VD at various doses are completely different in the middle-aged and old OVX females. It is likely that VD acts through a various mechanisms that are sensitive in female rats of old age with long-term absence of ovarian hormones. Moreover, it is completely needed to understand the precise mechanisms of how VD treatment alone or in a combination with 17β-estradiol supplementation may affect women’s anxiety-related state.

These points illustrate how the current state of VD treatment research is incomplete and in need of more intensive research. Working toward uncovering how the interaction between VD and estradiol changes after menopause, and the implications of these changes elsewhere in the postmenopausal woman, is necessary for providing the most complete understanding of how VD treatment alone or in a combination with 17β-estradiol supplementation may affect women’s affective-related state.

VD as changes in VDR impact on various brain neurotransmitters, and thus suggest a potential role of vitamin D in causing and redressing mood disorders [65]. It could be supposed, even though estrogens and cholecalciferol share similar targets on monoaminergic or another neurotransmitter systems to induce their anxiolytic-like effects, the behavioral manifestations of cholecalciferol are completely different in the old OVX females. It is likely that cholecalciferol acts through a different mechanisms of action that is sensitive to the age of female rats with long-term absence of ovarian hormones.

In conclusion, the results of this study can be summarized as follows: all tested doses of cholecalciferol given alone are produced anxiolytic-like effects in the EPM and LDT in the old OVX female rats; the one specific dose of cholecalciferol (1.0 mg/kg/day) in the old OVX rats is able to induce synergic anxiolytic-like effect in the EPM and LDT; effects of cholecalciferol on anxiety-like behavior in the old OVX rats after long-term absence of estrogen are dependent from absence or presence of additional hormonal treatment as 17β-E_2_. Further investigations is to be addressed in relation to such issues: whether different effects of cholecalciferol on anxiety-like behavior are dependent from different age of rats, or whether different doses of cholecalciferol on anxiety-like behavior in OVX rats with different age rats might lead to negative versus positive effects. Further research, with properly designed experimental studies, is needed to test this hypothesis. In addition, further research is needed to elucidate the biochemical mechanism/(s) of cholecalciferol effects on the anxiety-like behavior and their physiological relevance for development of mood impairment at estrogen deficiency at aging. Furthermore, the mechanism by which cholecalciferol produces anxiolytic-like effect in the old OVX rats and the implications of this in brain function need to be investigated in future research. Moreover, further studies are needed to evaluate the association of VD with estrogen-related pathways and to conduct detail experiments together with biochemical studies of these subjects to verify the significance of this study.

## Conclusions

The present data of the preclinical study indicates that chronic cholecalciferol at a dose of 1.0 mg/kg, s.c. treatment decreased anxiety-related behavior after impairment induced by long-term ovariectomy in the old female rats with long-term absence of estrogen. Moreover, a combination of cholecalciferol at a dose of 1.0 mg/kg s.c. and 17β-E_2_ is more effective than 17β-E_2_ alone in the old OVX rats inducing a more synergic anxiolytic-like effects in the EPM and LDT. Furthermore, this is the first study to show a beneficial effect of chronic cholecalciferol at dose of 1.0 mg/kg s.c. administration on anxiety-related states induced by long-term ovariectomy in the old female rats. Importantly, these results suggest that 17β-E_2_ administration interfered with anxiolytic-like action of cholecalciferol administered alone at doses of 2.5 mg/kg or 5.0 mg/kg to the old OVX rats with long-term absence of estrogens. This work promotes more effective creating of the novel therapeutic targets and strategies for anxiety-like treatment in the old subjects with long-term estrogen deficiency.
